# Potent Paracrine Effects of human induced Pluripotent Stem Cell-derived Mesenchymal Stem Cells Attenuate Doxorubicin-induced Cardiomyopathy

**DOI:** 10.1038/srep11235

**Published:** 2015-06-09

**Authors:** Yuelin Zhang, Xiaoting Liang, Songyan Liao, Weixin Wang, Junwen Wang, Xiang Li, Yue Ding, Yingmin Liang, Fei Gao, Mo Yang, Qingling Fu, Aimin Xu, Yuet-Hung Chai, Jia He, Hung-Fat Tse, Qizhou Lian

**Affiliations:** 1 Department of Medicine, Li Ka Shing Faculty of Medicine, the University of Hong Kong, Hong Kong; 2 Department of Biochemistry, Li Ka Shing Faculty of Medicine, the University of Hong Kong, Hong Kong; 3 Department of Ophthalmology, Li Ka Shing Faculty of Medicine, the University of Hong Kong, Hong Kong; 4 Otorhinolaryngology Hospital, The First Affiliated Hospital of Sun Yat-sen University, Guangzhou, China; 5 Shenzhen Institutes of Research and Innovation, the University of Hong Kong, China

## Abstract

Transplantation of bone marrow mesenchymal stem cells (BM-MSCs) can protect cardiomyocytes against anthracycline-induced cardiomyopathy (AIC) through paracrine effects. Nonetheless the paracrine effects of human induced pluripotent stem cell-derived MSCs (iPSC-MSCs) on AIC are poorly understood. *In vitro* studies reveal that doxorubicin (Dox)-induced reactive oxidative stress (ROS) generation and cell apoptosis in neonatal rat cardiomyocytes (NRCMs) are significantly reduced when treated with conditioned medium harvested from BM-MSCs (BM-MSCs-CdM) or iPSC-MSCs (iPSC-MSCs-CdM). Compared with BM-MSCs-CdM, NRCMs treated with iPSC-MSCs-CdM exhibit significantly less ROS and cell apoptosis in a dose-dependent manner. Transplantation of BM-MSCs-CdM or iPSC-MSCs-CdM into mice with AIC remarkably attenuated left ventricular (LV) dysfunction and dilatation. Compared with BM-MSCs-CdM, iPSC-MSCs-CdM treatment showed better alleviation of heart failure, less cardiomyocyte apoptosis and fibrosis. Analysis of common and distinct cytokines revealed that macrophage migration inhibitory factor (MIF) and growth differentiation factor-15 (GDF-15) were uniquely overpresented in iPSC-MSC-CdM. Immunodepletion of MIF and GDF-15 in iPSC-MSCs-CdM dramatically decreased cardioprotection. Injection of GDF-15/MIF cytokines could partially reverse Dox-induced heart dysfunction. We suggest that the potent paracrine effects of iPSC-MSCs provide novel “cell-free” therapeutic cardioprotection against AIC, and that MIF and GDF-15 in iPSC-MSCs-CdM are critical for these enhanced cardioprotective effects.

Anthracyclines, including doxorubicin (Dox), are common chemotherapeutic agents used for the treatment of a wide variety of malignancies^1^. Nonetheless they are well known to cause dose-dependent, progressive myocardial damage that can manifest to variable degrees, from subclinical myocardial dysfunction to severe heart failure and even death^2^. The prevalence of anthracycline-induced cardiomyopathy (AIC) is increasing as more cancer patients survive, and is a potential cause of significant morbidity and mortality^3^. Although pharmacological therapies, including the use of angiotensin converting enzyme inhibitors^4^,^5^ alone or with beta-blockers, may limit AIC, the only curative therapy for severe AIC is heart transplantation^6^. Recently, stem cell based therapy has been investigated^7^,^8^,^9^ as treatment for cardiovascular diseases, including AIC.

Among the different types of stem cell under investigation, bone marrow (BM) derived mesenchymal stem cells (MSCs)(BM-MSCs) show promising results in the treatment of heart failure with several advantages, including easy isolation and expansion and low immunogenicity^10^,^11^. The majority of the therapeutic effects of MSCs have been attributed to their paracrine effects^12^,^13^,^14^,^15^,^16^. MSCs can secrete a wide array of cytokines and growth factors for cardiac repair via modulation of the inflammatory response, inhibition of cell necrosis and apoptosis, and promotion of angiogenesis^17^,^18^. Although MSCs derived from different tissues share many common properties, they also display distinct paracrine potential. MSCs can be derived from either adult somatic tissue or pluripotent stem cells. Currently, BM-MSCs are the most common cell source investigated in clinical trials, although their limited proliferative capacity and large variability have hindered their application^19^,^20^. We have successfully derived functional MSCs from human embryonic stem cells (ESCs) and induced pluripotent stem cells (iPSCs): both exhibit superior therapeutic efficacy and paracrine actions for cardiovascular repair to BM-MSCs^21^,^22^,^23^. Unfortunately the potential risk of tumor formation and poor cell engraftment remain a major hurdle to the therapeutic application of MSCs derived from pluripotent stem cells. Nevertheless the administration of MSC-derived conditioned medium (CdM) that contains different cytokines can address these issues although the potential therapeutic benefits are unclear. In this study, we sought to investigate whether CdM derived from iPSCs-MSCs can confer a therapeutic effect in AIC.

Here, we demonstrate that human iPSC-MSCs-CdM effectively attenuates Dox-induced cardiomyopathy in mice by prevention of cardiomyocyte apoptosis and reduction of reactive oxidative stress (ROS) generation. In addition, our results reveal that iPSC-MSCs-CdM enriched with growth differentiation factor-15 (GDF-15) and macrophage migration inhibitory factor (MIF) provides better cardioprotection against AIC than BM-MSCs-CdM.

## Results

### iPSC-MSCs-CdM effectively reduces Dox-induced oxidative stress and apoptosis *in-vitro*

**Figure 1:**
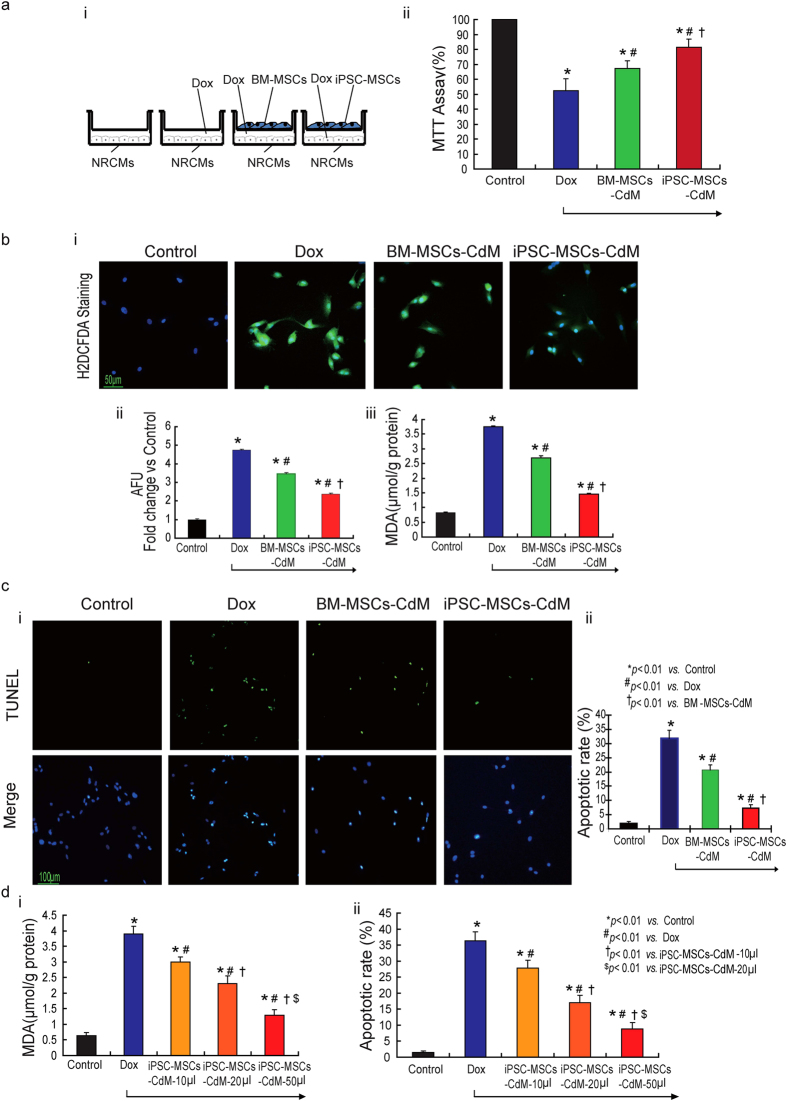
Paracrine effects of MSCs on Dox-induced oxidative stress and apoptosis of neonatal rat cardiomyocytes (NRCMs) *in-vitro*. **a**) 1 × 10^4^ iPSC-MSCs or BM-MSCs were seeded on 0.3 μm pore size transwell inserts and 1 × 10^4^ NRCMs were seeded on the bottom of a 96-well culture plate with or without presence of 1 μM Dox. 24 hours later, the transwell inserts were removed and MTT assay was used to measure NRCMs cell viability (**i**). The percentage of cell viability of each group was calculated from triple experiments (**ii**) (*p *<* *0.01* *vs*. control group; ^*#*^*p* *<* *0.01* *vs*. Dox group; ^†^*p* *<* *0.01 vs*. BM-MSCs-CdM group). **b**,**c**) NRCMs were treated with or without MSCs-CdM (BM-MSCs-CdM or iPSC-MSCs-CdM) under challenge of 1 μM Dox for 24 hours. In living cell culture conditions, H2DCFDA (**b**-**i**) and TUNEL (**c**-**i**) staining were used to measure ROS generation and cell apoptosis respectively. Quantitative measurement of ROS (**b**-**ii**) and apoptotic rate (**c**-**ii**) was counted from five viewing fields in each group and triple experiments were performed (*p *<* *0.01* *vs*. control group; ^*#*^*p* *< 0.01* *vs*. Dox group; ^†^*p* *<* *0.01 vs*. BM-MSCs-CdM group). The concentration of MDA (b-iii) among the different groups was also measured (*p *<* *0.01* *vs*. control group; ^*#*^*p* *<* *0.01* *vs*. Dox group; ^†^*p* *<* *0.01 vs*. BM-MSCs-CdM group). **d**) The effect of iPSC-MSCs-CdM showed a dose-dependent manner in attenuation of ROS generation and apoptosis induced by Dox. The concentration of MDA (**d**-**i**) and apoptosis (**d**-**ii**) among the different groups were also measured among the different groups was measured (*p *<* *0.01* *vs*. control group; ^*#*^ *p* *<* *0.01* *vs*. Dox group; ^†^*p* *<* *0.01 vs*. iPSC-MSCs-CdM-10 μl group; ^$^*p* *<* *0.01 vs*. iPSC-MSCs-CdM-20 μl group).

We sought to determine whether soluble factors of MSCs play a key protective role against Dox-induced cardiomyopathy. Neonatal rat cardiomyocytes (NRCMs) were cultured *in-vitro* with or without MSCs using a transwell to avoid direct cell-cell communication under challenge of Dox for 24 hours (Fig. 1a-i). 3-(4,5-cimethylthiazol-2-yl)-2,5-diphenyl tetrazolium bromide (MTT) assay demonstrated that co-culture with BM-MSCs or iPSC-MSCs significantly increased cell viability of NRCMs compared with the absence of MSCs (Dox group, Fig. 1a-ii). In addition, iPSC-MSCs exhibited better protective potential against Dox-induced NRCM death than BM-MSCs (Fig. 1a-ii, *P* *<* *0.01*).

Excessive ROS generation and cardiomyocyte apoptosis have been proposed as the major mechanisms of AIC. We further investigated whether the cardioprotective effects of MSCs-CdM are mediated through attenuation of Dox-induced oxidative stress and apoptosis in NRCMs. In living cell culture of NRCMs, 2′, 7′-Dichlorodihydrofluorescein dilacerate (H_2_DCFDA) and terminal deoxynucleotidal transferase mediated dUTP nick end-labeling (TUNEL) staining was used to measure ROS generation and apoptosis, respectively, in the presence or absence of MSCs-CdM under challenge of Dox for 24 hours (Fig. 1b,c). H_2_DCFDA staining revealed that the ROS score was significantly increased approximately five fold in the Dox group compared with the control group (Fig. 1b-i,ii). Administration of CdM derived from BM-MSCs or iPSC-MSCs significantly decreased the fluorescence intensity of H_2_DCFDA staining compared with the Dox group (Fig. 1b-ii; *P* *<* *0.01*). Similarly, the concentration of malonyldialdehyde (MDA) in the Dox group was dramatically increased compared with the control group (Fig. 1b-iii, 3.76 ± 0.015 μmol/g protein *vs*. 0.83 ± 0.014 μmol/g protein; *P* *<* *0.01*). Administration of CdM derived from BM-MSCs or iPSC-MSCs significantly decreased MDA compared with the Dox group (Fig. 1b-iii; *P* *<* *0.01*).

TUNEL staining revealed that apoptosis of NRCMs in the Dox group was significantly increased compared with the control group (Fig. 1c-i,ii, 31.75 ± 3% *vs*. 1.75 ± 0.9%; *P* *<* *0.01*). Administration of CdM derived from BM-MSCs or iPSC-MSCs significantly decreased apoptosis of NRCMs compared with the Dox group (Fig. 1c-ii; *P* *<* *0.01*). Notably, both the fluorescence intensity of H_2_DCFDA staining (Fig. 1b-ii; *P* *<* *0.01*) and the apoptotic rate of NRCMs (Fig. 1c-ii, 7.25 ± 1.25% *vs*. 20.75 ± 1.7%; *P* *<* *0.01*) were significantly lower following administration of iPSC-MSCs-CdM than following BM-MSCs-CdM. The concentration of MDA in the iPSC-MSCs-CdM group was also significantly lower than in the BM-MSC-CdM group (Fig. 1b-iii, 1.45 ± 0.04 μmol/g protein *vs*. 2.69 ± 0.07 μmol/g protein; *P* *<* *0.01*). The cardioprotection afforded by iPSC-MSCs-CdM was dose-dependent (Fig. 1d). Concentration of MDA generation was decreased with increased concentration of iPSC-MSCs-CdM treatment in NRMCs (Fig. 1d-i; *P* *<* *0.01*). Compared with the iPSC-MSCs-CdM-10 μl and iPSC-MSCs-CdM-20 μl group, concentration of MDA was most remarkably reduced in the iPSC-MSCs-CdM-50 μl group (Fig. 1d-i; *P* *<* *0.01*). Similarly, the Dox-induced apoptotic rate of NRCMs was dramatically attenuated in the iPSC-MSCs-CdM-50 μl group, compared with the iPSC-MSCs-CdM-10 μl and iPSC-MSCs-CdM-20 μl group (Fig. 1d-ii; *P* *<* *0.01*).

These findings suggest that soluble factors secreted by MSCs in the CdM are likely to confer important protective effects against Dox-induced oxidative stress and apoptosis in NRCMs via a paracrine fashion. The cardioprotective effects of CdM derived from iPSC-MSCs are superior to those derived from BM-MSCs.

### Administration of iPSC-MSCs-CdM attenuates Dox-induced cardiomyopathy in mice

**Figure 2:**
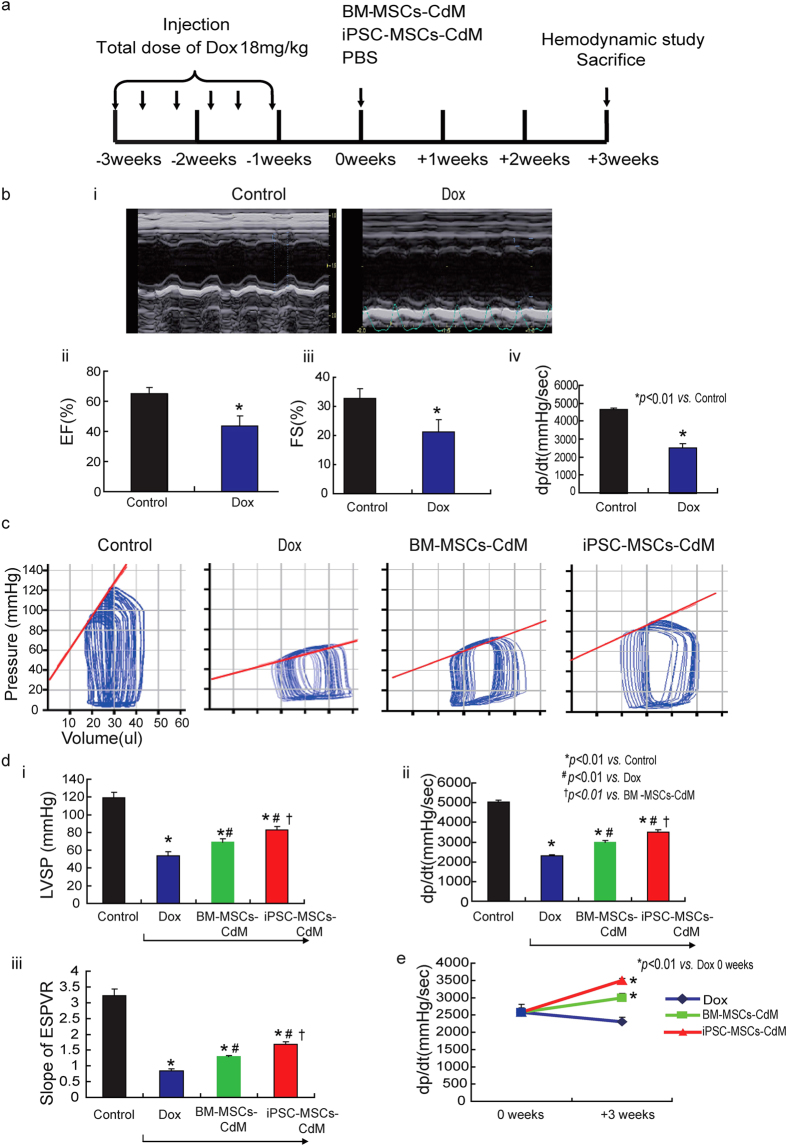
iPSC-MSCs-CdM transplantation attenuates Dox-induced cardiomyopathy in mice. **a**) Schematic chart showing induction of Dox-induced cardiomyopathy in mice and transplantation of BM-MSC-CdM or iPSC-MSC-CdM. **b**) Representative echocardiography photographs showing heart function in Control and Dox group at 0 week (**i**). Compared with control group, left ventricular ejection fraction (LVEF), fractional shortening (FS) and +dP/dt were dramatically reduced in the Dox group (**ii**,**iii**,**iv**) (*p *<* *0.01* *vs*. Control group). **c**) Representative PV-Loop photographs showing heart function in different groups at 3 weeks. **d**) Effects of BM-MSCs-CdM or iPSC-MSCs-CdM on left ventricular (LV) function assessed 3 weeks after CdM injection using cardiac catheterization. Left ventricular systolic pressure (LVSP); End systolic pressure volume relationship (ESPVR)(**ii**); and +dP/dt (**iii**) (*p *<* *0.01* *vs*. Control group; ^*#*^*p* *<* *0.01* *vs*. Dox group; ^†^*p* *<* *0.01 vs*. BM-MSCs-CdM group). **e**) J curve showing +dp/dt among different groups at 0 weeks and 3 weeks respectively (*p *<* *0.01* *vs*. Dox 0 weeks).

The animal experimental protocol is outlined in Fig. 2a. As shown in Fig. 2b, left ventricular ejection fraction (LVEF), fractional shortening (FS) and +dp/dt were significantly decreased in the Dox group at week 0 compared with the control group. This confirmed successful establishment of an animal model of Dox-induced cardiomyopathy (Fig. 2b; *P* *<* *0.01*). At 3-weeks, left ventricular systolic pressure (LVSP), +dp/dt and slope of end systolic pressure volume relationship (ESPVR) were significantly decreased in the Dox group compared with the control group (Fig. 2c,d-i–iii; *P* *<* *0.01*). In contrast, LVSP, +dp/dt and slope of ESPVR were significantly improved in the BM-MSCs-CdM group and iPSC-MSCs-CdM group at 3-weeks compared with the Dox group (Fig. 2c,d-i–iii; *P* *<* *0.01*). LVSP, +dp/dt and slope of ESPVR were also significantly higher in the iPSC-MSCs-CdM group than the BM-MSCs-CdM group (Fig. 2d-i–iii; *P* *<* *0.01*), indicating that iPSC-MSCs-CdM better aids recovery of heart function in AIC. Notably, both BM-MSCs-CdM and iPSC-MSCs-CdM groups, but not the Dox group, showed significant improvement in +dp/dt at 3-weeks compared with week 0 (Fig. 2e; *P* *<* *0.01*).

**Figure 3:**
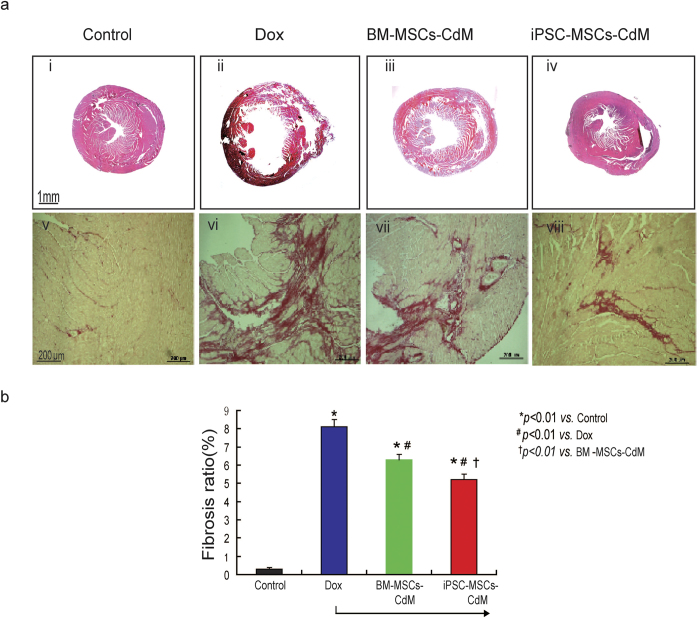
iPSC-MSCs-CdM transplantation reverses Dox-induced histological change in mice. **a**) At 3 weeks after CdM injection, histological examination with Hematoxylin and eosin staining for heart tissue among different groups (**i**–**iv**). Gross view of heart tissue among Control (**i**), Dox treated (**ii**), BM-MSCs-CdM (**iii**) and iPSC-MSCs-CdM (**iv**) treated mice. Compared with control mice (**i**), enlarged left ventricular cavity in doxorubicin treated mice was observed (**ii**). Sirius red staining showed a different myocardium fibrosis among experimental groups (**v**–**viii**). **b**) Quantitative measurement of fibrosis ratio of heart among different experimental groups (*p *<* *0.01* *vs*. Control group; ^#^*p* *<* *0.01* *vs*. Dox group; ^†^*p* *<* *0.01 vs*. BM-MSCs-CdM group).

Hematoxylin and eosin (HE) staining revealed a dilated LV chamber, the typical feature of AIC, in the Dox group (Fig. 3a-ii) compared with the control group (Fig. 3a-i). Nonetheless injection of both BM-MSCs-CdM and iPSC-MSCs-CdM significantly attenuated LV dilatation induced by Dox (Fig. 3a-iii,iv).

Cardiac fibrosis was assessed by Sirius red staining (Fig. 3a-v–viii). The ratio of cardiac fibrosis was greatly increased in the Dox group compared with the control group (Fig. 3b, 8.1 ± 0.4% vs. 0.3 ± 0.08%; *P* *<* *0.01*). This ratio of cardiac fibrosis was significantly decreased by 22.2% in the BM-MSCs-CdM group and by 36.8% in the iPSC-MSCs-CdM group compared with the Dox group (*P* *<* *0.01*). Notably, the ratio of cardiac fibrosis was also significantly lower in the iPSC-MSCs-CdM group than the BM-MSC-CdM group (5.1 ± 0.3% *vs*. 6.4 ± 0.3%; *P* *<* *0.01*), indicating that iPSC-MSCs-CdM is superior to BM-MSCs-CdM in attenuation of Dox-induced cardiac fibrosis (Fig. 3b).

These results demonstrate that injection of BM-MSCs-CdM or iPSC-MSCs-CdM significantly improves LV function and decreases myocardial fibrosis in a mouse model of Dox-induced cardiomyopathy. In addition, iPSC-MSCs-CdM is more effective than BM-MSCs-CdM.

### Attenuated Dox-induced cardiomyopathy of mice is linked to reduced oxidative stress and cardiomyocyte apoptosis *in-vivo*

**Figure 4:**
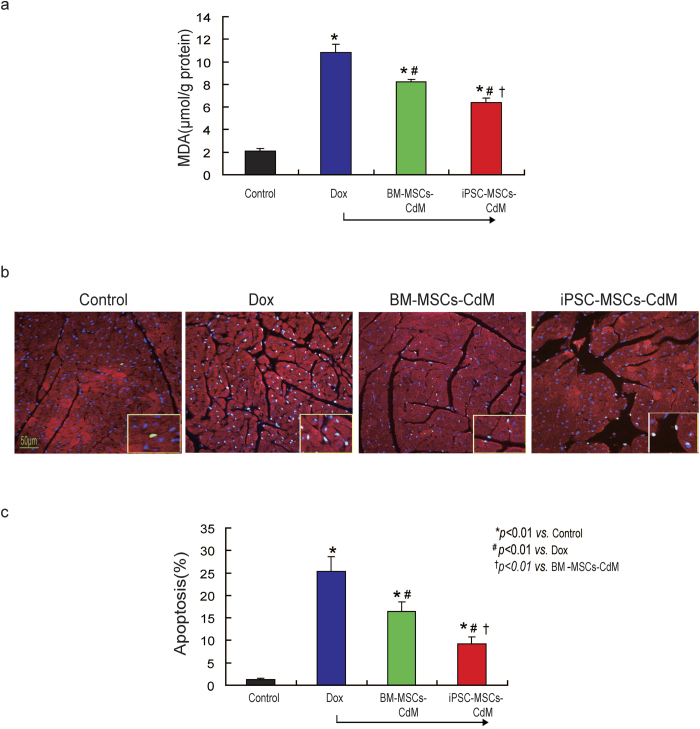
iPSC-MSCs-CdM transplantation ameliorates ROS generation and apoptosis of cardiomyocytes induced by Dox in the heart tissue of mice. **a**) At 3 weeks after CdM injection, the concentration of MDA among different groups was measured (*p *<* *0.01* *vs*. Control group; ^#^*p* *<* *0.01* *vs*. Dox group; ^†^*p* *<* *0.01 vs*. BM-MSCs-CdM group, n = 3). **b**) Representative photographs showing differential accumulation of TUNEL positive cells among Control, Dox, BM-MSCs-CdM treatment (BM-MSCs-CdM), or iPSC-MSCs-CdM treatment (iPSC-MSCs-CdM) group. **c**) Quantitative measurement of the apoptotic rate of myocardium was expressed as percent of positive TUNEL cells vs. total DAPI positive cells per viewing area (*p *<* *0.01* *vs*. Control group; ^*#*^*p* *<* *0.01* *vs*. Dox group; ^†^*p* *<* *0.01 vs*. BM-MSCs-CdM group, n = 6).

The level of ROS in heart tissue among different groups was evaluated by MDA. As shown in Fig. 4a, compared with the control group, the concentration of MDA was dramatically increased in the Dox group (Fig. 4a, 2.1 ± 0.23 μmol/g protein *vs*. 10.8 ± 0.78 μmol/g protein; *P* *<* *0.01*). Nonetheless this concentration was significantly decreased by 24% in the BM-MSCs-CdM group and by 40% in the iPSC-MSCs-CdM group compared with the Dox group (*P* *<* *0.01*). The concentration of MDA was also significantly lower in the iPSC-MSCs-CdM group than in the BM-MSCs-CdM group (Fig. 4a, 6.4 ± 0.4 μmol/g protein *vs*. 8.2 ± 0.25 μmol/g protein; *P* *<* *0.01*).

Next, cell apoptosis in different groups was determined by TUNEL staining. Compared with the control group, cell apoptosis was significantly increased over the myocardium in the Dox group (Fig. 4b,c, 25.4 ± 3.2% vs. 1.22 ± 0.3%; *P* *<* *0.01*). Administration of BM-MSCs-CdM or iPSC-MSCs-CdM significantly decreased the cell apoptotic rate (Fig. 4b,c; *P* *<* *0.01*) over the myocardium compared with the Dox group. The cell apoptotic rate was much lower in the iPSC-MSC-CdM group than in the BM-MSCs-CdM group (Fig. 4b,c, 9.2 ± 1.5% vs.16.4 ± 1.2%; *P* *<* *0.01*). These findings indicate that iPSC-MSCs-CdM is more potent than BM-MSCs-CdM in reducing ROS and attenuating CMC apoptosis induced by Dox *in-vivo*.

### Distinct enrichment of paracrine components between iPSC-MSCs and BM-MSCs

To understand the differential paracrine capacity of BM-MSCs and iPSC-MSCs against Dox-induced cardiotoxicity, we performed a cytokine antibody array that covered 507 human cytokines/chemokines and growth factors in the serum free-CdM derived from BM-MSCs and iPSC-MSCs culture. Both BM-MSCs and iPSC-MSCs were able to release a wide spectrum of cytokines, chemokines, adipokines, growth factors, angiogenic factors and soluble receptors.

Pair-wise comparison of the protein profiling of CdM revealed many common characteristics but also some distinct differences. Overall, 214 of 507 factors were commonly detected in both BM-MSCs and iPSC-MSCs with <2.0-mean fold difference and *P>0.1* from two tailed paired t-test (no significant difference). Classification of these factors into bioprocesses disclosed that these secreted factors were involved in metabolism, immunity, apoptosis, migration and differentiation as well as homeostasis. Nonetheless 13 factors were preferentially over-represented in BM-MSCs (mean fold difference *>2.0* and *P* *<* *0.1*); and 129 of 507 protein factors were preferentially over-represented in iPSC-MSCs (mean fold difference *>2.0* and *P* *<* *0.1*).

**Figure 5:**
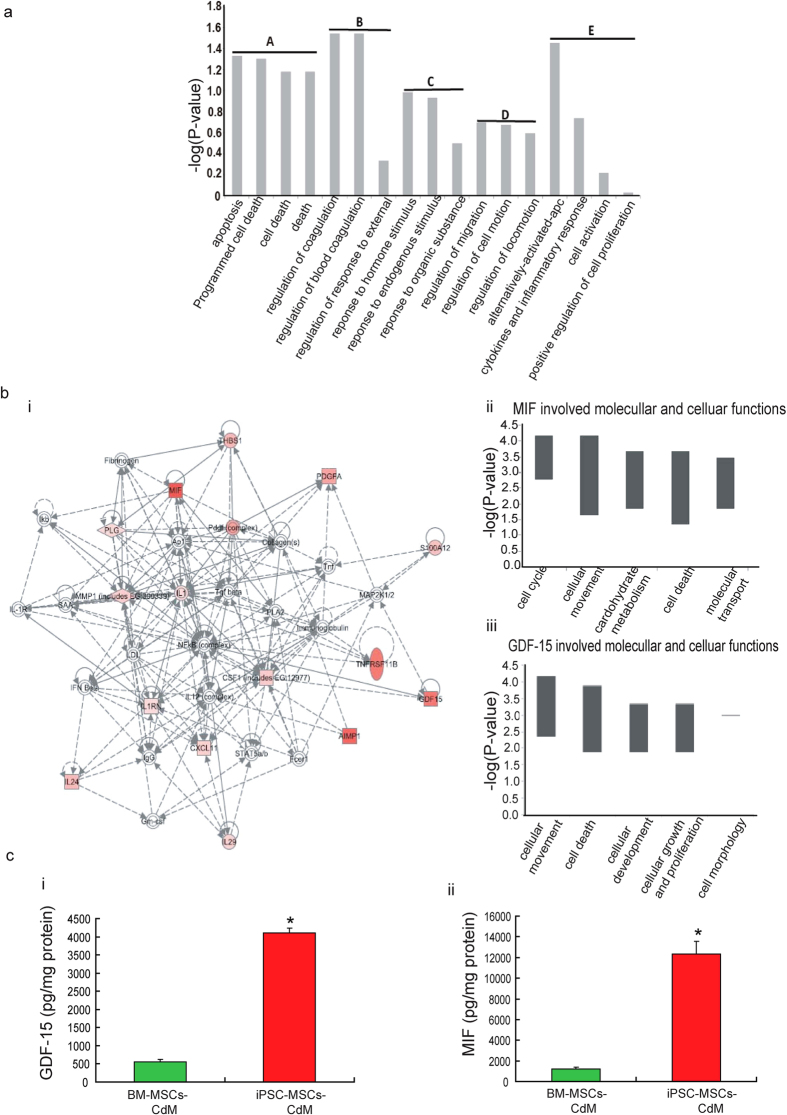
Profile of paracrine factors in the conditioned medium from iPSC-MSCs. **a**) Functional classification of the top 20 factors preferentially over-presented in iPSC-MSC-CdM. Here the p-value, or called EASE score, is a modified one-tail Fisher exact probability value used for gene-enrichment analysis in DAVID system. Each gene group has been assigned several annotation terms, and each gene group’ enrichment score stands for the geometric mean of negative denary logarithm of EASE scores of those terms involved in this group. Groups with enrichment score higher than 0.6 were shown in the figure (Enrichment score for group A: 1.25; B: 1.14; C: 0.81; D: 0.65; E: 0.61). **b**) Interactions and functional network identified by IPA. Genes in red indicate over-expressed genes found in iPSC-MSCs secretion compared with BM-MSC secretion. Deeper color means higher fold ratio for iPSC-MSCs- compared with BM-MSCs-secretion (**i**). MIF involved molecular and cellular functions identified by IPA. Ranged p-values are shown in negative denary logarithm form in y-axis (**ii**). GDF-15 involved molecular and cellular functions identified by IPA. Ranged p-values are shown in negative denary logarithm form in y-axis (**iii**). **c**) The concentration of GDF-15 among different groups was measured (**i**) (*p *<* *0.01* *vs*. BM-MSCs-CdM group, n = 3). The concentration among different groups was measured (**iii**) (*p *<* *0.01* *vs*. BM-MSCs-CdM group, n = 3).

Our results revealed that most prominent distinctions preferentially found in iPSC-MSCs secretions were closely associated with regulation of apoptosis, response to exogenous and endogenous stimulus, cell migration, inflammatory response and cell activation (Table 1). The top 20 protein factors over-represented in iPSC-MSCs-CdM were classified into biological processes using the NIH DAVID Pathway Analysis tool (Fig. 5a). Although we do not know the functional role of each cytokine/growth factor or which play the most important role in myocardial repair, many of these factors have been individually shown to have positive effects on cardioprotection and to be involved in anti-inflammation (GDF-15^24^, MIF), anti-apoptosis (endothelial monocyte-activating polypeptide-II, Thrombospondin-1), anti-oxidation (MIF), and pro-proliferation and migration of cardiac progenitor cells and cardiomyocytes (platelet derived growth factor AA, erythoblastic leukemia viral oncogene homolog 3). Ingenuity pathway analysis software identified that the functional networks of the top 20 factors were involved in modulation of cell survival (e.g. nuclear factor kappa B signaling), stress response (e.g. mitogen-activated protein kinase 1/2 signaling), extracellular matrix metabolism (e.g. matrix metallopeptidase 1, collagens, fibronectin), immunomodulation (e.g. transforming growth factor belta signaling, immunoglobulin) and inflammation (e.g. interleukinI-1, interleukin-12 signaling) (Fig. 5b-i). Additional analysis revealed that the cellular and molecular functions of MIF and GDF-15 factors were significantly associated with cellular movement, cell cycle, cell metabolism and cell death, cellular development and cellular growth, all important for promoting myocardial regeneration (Fig. 5b-ii,iii).

Consistent with the cytokine array results, the concentration of GDF-15 and MIF in iPSC-MSCs-CdM and BM-MSCs-CdM was measured by Enzyme-linked Immunosorbent Assay (ELISA) assay. As shown in Fig. 5c, the concentration of GDF-15 (Fig. 5c-i, 4113 ± 121 pg/mg protein vs. 559 ± 55 pg/mg protein; *P* *<* *0.01*) and MIF (Fig. 5c-ii, 12316 ± 1244 pg/mg protein vs. 1239 ± 151 pg/mg protein; *P* *<* *0.01*) was significantly higher in iPSC-MSCs-CdM than in BM-MSCs-CdM.

In contrast, the functional enrichment profiles of the secreted factors over-represented in BM-MSCs-CdM (Table 2) were more dispersed. Interestingly, the enrichment score for the apoptosis group in BM-MSCs-CdM was 5-fold lower compared with iPSC-MSCs-CdM (enrichment score 0.24 *versus* 1.25), although the enrichment score for the cell migration group was similar (enrichment score 0.82 *versus* 0.65). These observations demonstrate a differential profile of secretions for BM-MSCs and iPSC-MSCs that might account for their different therapeutic effect in Dox-induced cardiomyopathy. Bioinformatics analysis has revealed that iPSC-MSCs secretions are preferentially involved in anti-apoptosis, anti-inflammation, regulation of cell mobilization and cell proliferation compared with BM-MSCs secreted factors.

### Enriched GDF-15 and MIF in iPSC-MSCs secretome contributes to attenuation of Dox-induced ROS and apoptosis of cardiomyocytes

**Figure 6:**
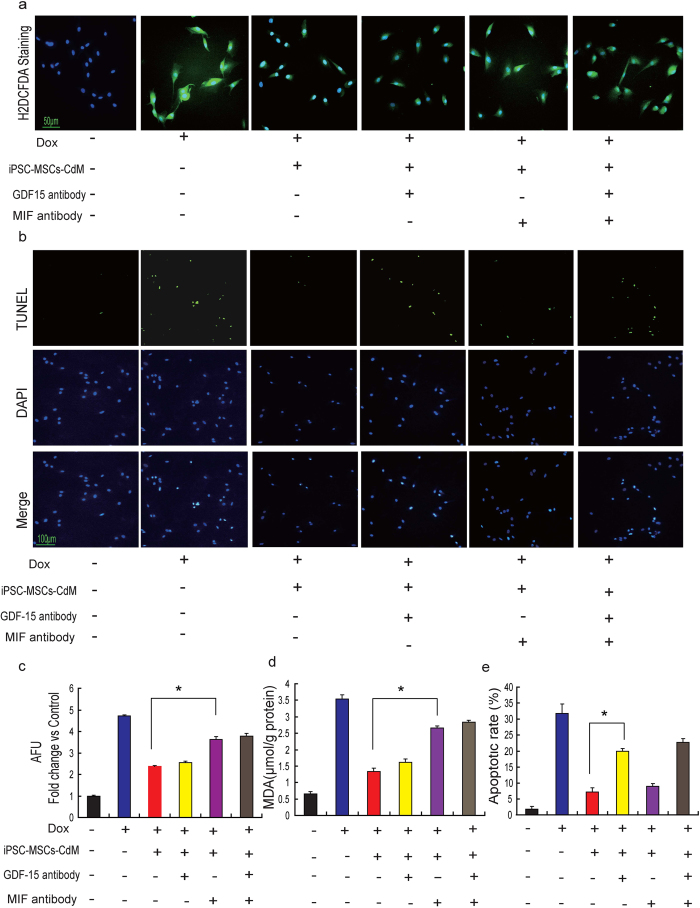
Role of MIF and GDF-15 in iPSC-MSC-CdM-attenuated Dox-induced cardiotoxity. **a**) Representative photographs showing ROS generation among Control, doxorubicin (Dox), iPSC-MSCs-CdM-control antibody treatment (iPSC-MSCs-CdM-ConAb), or MIF and/or GDF-15 depleted iPSC-MSCs-CdM treatment. **b**) Representative photographs showing the apoptosis of NRCMs among Control, doxorubicin (Dox), iPSC-MSCs-CdM -control antibody treatment (iPSC-MSCs-CdM-ConAb), or MIF and/or GDF-15 depleted iPSC-MSCs-CdM treatment. **c**) Quantitative measurement of immunofluorescent intensity of ROS among different experimental groups after normalized to control group (*p *<* *0.01*, n = 3). **d**) The concentration of MDA among different groups was measured (*p *<* *0.01*, n = 3). **e**) Quantitative measurement of apoptotic rate of NRCMs among different experimental groups. Quantitative measurement of ROS and apoptotic rate was counted from five viewing fields in each group and triple experiments were performed (*p *<* 0.01).

To explore whether GDF-15 and MIF are responsible for the enhanced therapeutic effects of iPSC-MSCs-CdM, we investigated the effects of GDF-15- or MIF-depleted iPSC-MSCs-CdM on Dox-induced cardiotoxicity. Both GDF-15 and MIF were depleted from iPSC-MSCs-CdM by immunoprecipitation with antibodies specific for GDF-15 and MIF, respectively. After depletion, the concentration of GDF-15 was reduced from 4113 ± 121 pg/mg protein to 90 ± 10 pg/mg; while MIF was reduced from 12316 ± 1244 pg/mg protein to 120 ± 16 pg/mg; suggesting the immunodepletion of GDF-15 and MIF was successful. As shown in Fig. 6a,c, iPSC-MSCs-CdM-inhibited ROS generation was remarkably attenuated by depletion of MIF from iPSC-MSCs-CdM (Fig. 6a,c; *P* *<* *0.01*). The reduction in NRCM apoptosis by iPSC-MSCs-CdM was partly attenuated by depletion of GDF-15 from iPSC-MSCs-CdM (Fig. 6b,e; *P* *<* *0.01)*. In addition, the concentration of MDA was significantly increased by depletion of MIF from iPSC-MSCs-CdM compared with iPSC-MSCs-CdM (Fig. 6d; *P* *<* *0.01*). This indicates that MIF and GDF-15 in iPSC-MSCs-CdM contribute to the reduced ROS generation and attenuation of NRCM apoptosis.

### Immunodepletion of MIF and GDF-15 reduces the efficiency of iPSC-MSCs-CdM to rescue Dox-induced cardiomyopathy

**Figure 7:**
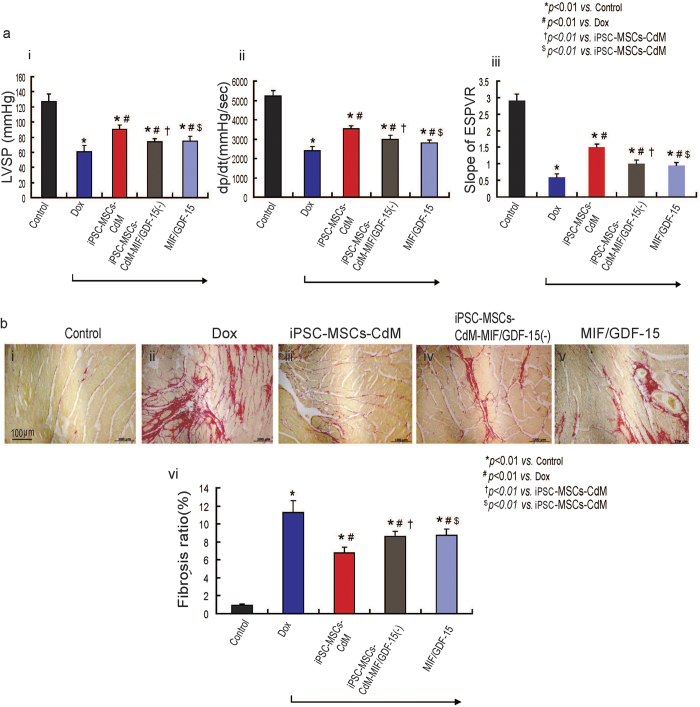
Immunodepletion of MIF and GDF-15 in iPSC-MSCs-CdM fails to rescue the Dox-induced cardiomyopathy. **a**) Different cardioprotective effects of iPSC-MSCs-CdM or iPSC-MSCs-CdM-MIF/GDF-15(−) or MIF/GDF-15 transplantation on LV geometry and function, including LVSP (**i**), +dp/dt (**ii**) and ESPVR (**iii**) assessed 3 weeks after CdM injection using cardiac catheterization (*p *<* *0.01* *vs*. Control group; ^#^*p* *<* *0.01* *vs*. Dox group; ^†^*p* *<* *0.01 vs*. iPSC-MSCs-CdM group; ^$^*p* *<* *0.01* vs. iPSC-MSCs-CdM group n = 6). b) Sirius red staining showing a different myocardium fibrosis among Control group (**i**); Dox group (**ii**); iPSC-MSCs-CdM (**iii**) group; iPSC-MSCs-CdM-MIF/GDF(−) group (**iv**); MIF/GDF-15 (**v**). Quantitative measurement of fibrosis of heart among different experimental groups (**v**) (*p *<* *0.01* *vs*. Control group; ^#^*p* *<* *0.01* *vs*. Dox group; ^†^*p* *<* *0.01 vs*. iPSC-MSCs-CdM group, n = 6; ^$^*p* *<* *0.01* vs. iPSC-MSCs-CdM group n = 6).

To verify the role of MIF and GDF-15 in iPSC-MSCs-CdM for the treatment of Dox-induced cardiomyopathy, we compared the cardioprotective effects of iPSC-MSCs-CdM with immunodepletion of MIF and GDF-15 in iPSC-MSCs-CdM in a mouse model of AIC. Three weeks following CdM injection, the LVSP, LV +dp/dt and ESPVR were remarkably reduced in the iPSC-MSCs-CdM-MIF/GDF-15(−) group compared with the iPSC-MSCs-CdM group (Fig. 7a-i–iii; *P* *<* *0.01*). Sirius Red staining also demonstrated that fibrosis was much higher in the iPSC-MSCs-CdM-MIF/GDF-15(−) group compared with the iPSC-MSCs-CdM (Fig. 7b-i–iv,vi; *P* *<* *0.01*).We also examined the effects of recombinant MIF and GDF-15 with the same concentration of MIF/GDF-15 in iPSC-MSC-CdM on heart function in a mouse model of AIC. Three weeks following MIF/GDF-15 injection, LVSP, LV +dp/dt and ESPVR were significantly higher in the MIF/GDF-15 treated group than in the Dox group (Fig. 7a-i–iii; *P* *<* *0.01*). Sirius Red staining showed that the fibrosis ratio was markedly reduced in the MIF/GDF-15 treated group compared with the Dox group (Fig. 7b-i–vi; *P* *<* *0.01*). There was also a significant difference between iPSC-MSCs-CdM and MIF/GDF-15 in heart function or fibrosis ratio (Fig. 7a,b; *P* *<* *0.01*). These results indicate that enrichment of iPSC-MSCs-CdM with MIF and GDF-15 contributed to cardioprotection against Dox-induced cardiomyopathy.

## Discussion

The findings of this study demonstrate that both human iPSC-MSCs-CdM and BM-MSCs-CdM provide cardioprotection against Dox-induced cardiomyopathy by reducing ROS and cardiomyocyte apoptosis. The therapeutic effects of iPSC-MSCs-CdM are more potent than those of BM-MSCs-CdM. In particular, iPSC-MSCs-CdM enriched with MIF and GDF-15 contributes to the reduced ROS generation and apoptosis of cardiomyocytes induced by Dox.

Although iPSC-MSCs and BM-MSCs share many common properties, iPSC-MSCs have several unique advantages such as their higher proliferative capacity and a more robust differentiation potential^22^. The potential risk of tumor formation and poor cell engraftment with iPSC based transplantation nonetheless remains a concern. As the majority of the proposed benefits of MSC-based therapy is attributed to their paracrine effects, rather than direct trans-differentiation^21^,^25^,^26^, this prompted us to compare the potential therapeutic effects of CdM derived from human BM-MSCs and iPSC-MSCs in the treatment of Dox-induced cardiomyopathy.

In this study, we confirmed that cardiomyocyte apoptosis induced by increased ROS generation is one of the major mechanisms of Dox-induced cardiomyopathy^27^,^28^,^29^. Consistent with our prior studies^23^,^30^, we demonstrated that compared with BM-MSCs-CdM, iPSC-MSCs-CdM provides stronger anti-oxidative stress and anti-apoptotic actions that contribute to superior therapeutic efficacy in attenuation of Dox-induced cardiomyopathy. Our findings from antibody array showed that iPSC-MSCs and BM-MSCs not only release similar cytokines but also many distinct paracrine factors that may account for the superior therapeutic effects of iPSC-MSCs-CdM. Analysis of the highly expressed cytokines of iPSC-MSCs-CdM showed that those secreted factors are involved in anti-apoptosis, anti-inflammation/oxidation, and regulation of cell mobilization and reactivation. Among them, we detected a >9.0-fold higher level of MIF that was over-represented in iPSC-MSCs-CdM compared with BM-MSCs-CdM. MIF confers potent cardioprotective effects by reducing oxidative stress^31^,^32^ and activates the cardioprotective AMP-activated protein kinase pathway. Similarly, a >7.5-fold higher level of GDF-15 was strikingly over-presented in iPSC-MSCs-CdM compared with BM-MSCs-CdM. GDF-15 has been recently identified as a critical cardioprotective factor with anti-apoptotic, anti-inflammatory and anti-cardiac remodeling properties in response to myocardial injury^33^. The therapeutic effects of iPSC-MSC-CdM are greatly abolished by neutralizing MIF and GDF-15. Insulin-like growth factor (IGF)-1, a well-known key factor that activates resident endogenous cardiac stem/progenitor cells^34^; and Neuropilin-2, a key factor that promotes cardiac progenitor cells (CPC) and endothelial cell survival and migration^35^, were also greater than two fold higher in iPSC-MSCs-CdM than in BM-MSCs-CdM.

As shown in the present study, a cytokine cocktail derived from MSC-derived CdM may provide a unique therapeutic tool for the treatment of AIC. Despite major advances in the prevention and treatment, such as the use of angiotensin converting enzyme inhibitors alone or with beta-blockers, AIC remains a major cause of morbidity and mortality in cancer survivors^36^. The use of iPSC-MSCs-CdM may be a novel therapeutic approach to prevent or treat AIC, especially in those in whom conventional medical therapy has failed. There is an emerging interest to apply MSC-derived secretome or exosome for organ repair and/or regeneration^21^,^37^. Recently, transplantation of stem cell-conditioned medium, instead of stem cells, has been shown to be effective for tissue/cell repair in a range of conditions such as ischemic heart disease^38^,^39^,^40^, retinal ischemia^41^, acute lung injury^42^, chemical-induced colitis, enteric neuropathy^43^ and spinal cord injury^44^. Delivery may be by a single (immediate or delayed) administration^38^,^40^,^41^,^43^ or continuous administration over a short period of time^39^,^44^. MSCs can release many beneficial cytokines that are important in cardiac protection, but they can also release potentially harmful factors^45^. Thus optimization of MSC paracrine factors is preferable in order to maximize their beneficial effects. In this study, we identified two crucial cytokines, GDF-15 and MIF, which are highly presented in the iPSC-MSCs-CdM for heart repair, that offer great potential for the future development of a therapeutic MSC-secreted cocktail. A similar approach may also be applied to optimize the paracrine profile of iPSC-MSCs-CdM for myocardial repair in different cardiovascular diseases.

There are many advantages of stem cell conditioned medium for tissue repair. Nonetheless in certain disease conditions, transplantation of stem cells may be superior as it enables stem cell differentiation and engraftment to replace damaged myocardial tissue^46^.

There are some limitations to this study. First, only the two most prominent cytokines in iPSC-MSCs-CdM secretion were analyzed. The functions of other factors that are significantly enriched in iPSC-MSCs-CdM need further investigation. Second, there is increasing evidence that paracrine factor profiling of MSCs is highly dependent on the microenvironment^30^,^47^. MSCs are sensitive to the inflammatory microenvironment and may polarize into two distinct phenotypes following specific toll like receptor stimulation, and result in different immune-modulatory effects and distinct secretions^48^. Polarization of at-rest MSCs into a pro-inflammatory phenotype (MSC1) or an immunosuppressive phenotype (MSC2) that responds to different stimulation provides an attractive model to understand the multiple facets of MSCs^49^. We harvested at-rest MSC secretion *in vitro* that may not truly reflect MSC-mediated myocardial repair *in vivo*. Third, the optimal dose of CdM for cardioprotection has not been determined. Fourth, the long-term impact of CdM on heart function and survival was not investigated in this study.

This study provides evidence that iPSC-MSCs-CdM is more effective than adult BM-MSCs-CdM for cardioprotection against Dox-induced cardiomyopathy. The distinct protective effect of iPSC-MSCs-CdM is associated with some enriched important cytokines such as MIF and GDF-15 that are involved in anti-apoptosis, anti-inflammation/oxidation, regulation of cell mobilization and reactivation. We have provided evidence that iPSC-MSCs-CdM may serve as a novel therapeutic approach for the future development of “cell-free” therapy for cardiovascular repair.

## Methods

### Cell culture

Characterized BM-MSCs from healthy adults were commercially acquired from Cambrex BioScience (Cat. No. PT-2501). Human iPSC-MSCs derived from iPSC lines have been previously described^22^. At least two iPSC-MSC lines (IMR90-iPSC-MSCs and Lee NL-iPSC-MSCs; passage 9 ~ 10) and two BM-MSC lines (passage 4 ~ 5) were used in this study. MSCs were cultured with Dulbecco’s Modified Eagle’s medium (DMEM) plus 10% fetal calf serum (GIBCO), basic fibroblast growth factor (bFGF, 5 ng/mL), and epidermal growth factor (EGF, 10 ng/mL). Cells were diluted at a ratio of 1:3 when they reached 70–80% confluence.

### Preparation of CdM and cytokine assays

As described previously^23^, CdM of cultured MSCs was prepared. In brief, MSCs were trypsinized and plated on a 15-cm plate, a total of 5 × 10^6^ cells. Twenty-four hours later, the regular culture medium was replaced with 15 ml of serum- and antibiotic-free DMEM (no EGF and bFGF). After a further 24 hours, the supernatant was aspirated gently, filtered through a 0.22 μm filter, then transferred to ultrafiltration conical tubes (Amicon Ultra-15 with membranes selective for −5 kDa), and finally centrifuged (4,000 *g* for 30 min at 4 °C) to concentrate the CdM. The final concentration was adjusted to 20 times that of the collected CdM.

Cytokine assay of MSC-CdM was performed using RayBio® Cytokine antibody array as previously described^21^. Briefly, a total of 200 μl of BM-MSCs-CdM or iPSC-MSCs-CdM was assayed for the presence of cytokines and other proteins according to the manufacturer’s instructions (RayBiotech, Norcross, GA. Cat No: AAH-BLG-1-4). Quantitative human cytokines were measured using customized Bio-Plex cytokine assay (Bio-Rad Laboratories, Hercules, CA; Cat No. M50007VNJK and MF00038C9E) according to the manufacturer’s instructions.

### Comparison of protein expression in iPSC-MSCs-CdM and BM-MSCs-CdM

The proteins in two iPSC-MSCs-CdMs and two BM-MSCs-CdMs were compared. According to the expression value, the proteins were be classified into three types: commonly detected in both BM-MSCs and iPSC-MSCs with <2.0-mean fold difference and *P> 0.1* from two tailed paired t-test, preferentially expressed in BM-MSCs (mean fold difference*>2.0* and *P* *<* *0.1*), or preferentially expressed in iPSC-MSCs (mean fold difference *>2.0* and *P* *<* *0.1*). In each group, gene-enrichment analysis and functional classifications were conducted by The Database for Annotation, Visualization and Integrated Discovery (DAVID) v6.7. Each protein group is associated with several annotation terms, and each term is coupled with a p-value, or so-called EASE score, which is a modified one-tail Fisher exact probability value. The protein groups’ enrichment score stands for the geometric mean of negative denary logarithm of EASE scores of those terms involved in this group. Interactions and the functional network centered on certain factors were identified by INGENUITY pathway analysis (IPA).

### NRCMs and culture

The NRCMs were isolated and cultured as described previously^50^. Briefly, hearts were quickly removed from neonatal Wistar rats (0- to 1-day-old) sacrificed by decapitation, rinsed four times with modified Hank’s solution, and cut into small pieces on ice. The tissue fragments were warmed in a 50 ml tube in a water bath with a magnetic bar for 10 min at 37 °C. After discarding the supernatant, the minced myocardium was digested with fresh pre-warmed 0.2% trypsin for 5 min at 37 °C, and then the supernatant was collected gently and transferred to a 50 ml tube on ice containing 7 ml fetal bovine serum. These two steps were repeated to collect all the supernatant that was then centrifuged at 156.8 g for 5 min to collect the cells. Cells were re-suspended in NRCM culture medium to reduce fibroblast contamination. Finally, the supernatant was aspirated gently, and the cells plated in 24-well plates containing collagen-coated glass coverslips at a density of 2 × 10^5^ cells/ml. Culture media was changed every day. NRCMs were assigned to four different culture conditions for 24 hours: 1) control group; 2) 1 μM Dox and 50 μl serum- and antibiotic-free DMEM; 3) 1 μM Dox and 50 μl BM-MSCs-CdM; and 4) 1 μM Dox and 50 μl iPSC-MSCs-CdM. We employed the same amount of total protein from iPSC-MSC-CdM and BM-MSC-CdM in the studies. A higher concentration of iPCS-MSC-CdM was adjusted with basal medium (Phenol free DMEM) for the same volume (50 μl) as BM-MSC-CdM (50 μl) before use. To assess the dose-dependent effect of iPSC-MSCs-CdM, NRCMs were assigned to four different culture conditions for 24 hours: control group; 1) 1 μM Dox and serum- and antibiotic-free DMEM; 2) 1 μM Dox and 10 μl iPSC-MSCs-CdM; 3) 1 μM Dox and 20 μl iPSC-MSCs-CdM; 4) 1 μM Dox and 50 μl iPSC-MSCs-CdM.

### Detection of ROS and apoptosis

To detect the generation of ROS induced by Dox, H_2_DCFDA staining was performed. Briefly, NRCMs were cultured in 24-well plates with collagen-coated glass coverslips, and treated as described above. Then NRCMs were incubated with H_2_DCFDA (10 μM; Invitrogen) for 10 min at 37 °C in the dark. Subsequently, cells were washed with PBS twice and mounted with 4′, 6-diamidino-2-phenylindole (DAPI). Fluorescence intensity for ROS signal (percent arbitrary fluorescence units, % AFU) was measured on 300 cells using Image J software. Finally, fluorescence intensity was calculated from five different view fields of each group in three independent experiments.

To demonstrate the protective effects of MSCs-CdM on Dox-induced apoptosis of NRCMs, TUNEL staining was performed. Cell samples were fixed with fixation solution, and incubated with blocking solution and then with permeabilisation solution. The heart sections were washed and incubated with 1 μg/ml Proteinase K/10 mM Tris solution for 15 min at room temperature and washed twice in PBS. Finally, all samples were incubated with 50 μl TUNEL reaction mixture in a humid chamber for 1 hour at room temperature. Sections were then washed 3 times in PBS, mounted with DAPI, observed under a fluorescent microscope and finally photographed.

### ELISA

According to the manufacturer’s instructions, the concentration of GDF-15 and MIF in the CdM from iPSC-MSCs and BM-MSCs was measured using a human GDF-15 Elisa kit (Human GDF-15 DuoSet, DY957, R&D systems) and human MIF Elisa kit (Human MIF DuoSet, DY289, R&D systems) respectively.

### Immunodepletion of GDF-15 and MIF from iPSC-MSCs-CdM

GDF-15 and MIF were depleted from iPSC-MSCs-CdM as described previously^51^. Briefly, 0.5 μg of anti-GDF-15 (Clone 147627; R&D) and/or anti-MIF antibodies (Clone12302; R&D) or normal human IgG control antibody (1-001-A; R&D) were mixed with a suspension (50% slurry) of protein G-agarose beads in PBS at 4 °C for 1 hour with intermittent shaking. Following recovery by centrifugation, beads were washed three times and used for immunodepletion of GDF-15 and MIF. iPSC-MSC-CdM (1 ml) was incubated with protein G-agarose beads immobilized with anti-GDF-15 and/or anti-MIF antibodies or control human antibody for 1 hour at 4 °C. Immune complexes absorbed on protein G-agarose beads were precipitated by centrifugation. Finally, iPSC-MSCs-CdM was collected and centrifuged to 50 μl and then used immediately.

### Animal model

All animal experiments were performed in accordance with relevant guidelines and regulations by the University of Hong Kong and approved by the Committee on the Use of Live Animals in Teaching and Research (CULTAR) (Approval ID:3358-14). The animal model of AIC was induced in adult mice (6–8 weeks, ICR strain) by intraperitoneal injection of Dox (3 mg/kg, 3 times per week with a total cumulative dose of Dox 18 mg/kg)^52^. In the negative control group, mice were injected with an equal volume of saline. To confirm the successful creation of an animal model of AIC, echocardiography and invasive hemodynamic assessment were performed to measure heart function (n = 6) one week following completion of Dox injection. Since a single direct intramyocardial injection of 100 μl of MSC-CdM into rat heart^53^, or 50 μl of adipose-derived stem cell (ADSC)-CdM into mice heart had been reported to improve ischemic heart function^54^, we decided to inject 50 μl of MSCs-CdM into mice heart in this study. A further group of mice with AIC were randomized to receive intramyocardial injection of 1) phosphate-buffered saline (Dox group, n = 16); 2) 50 μl BM-MSCs-CdM (BM-MSCs-CdM group, n = 13); or 3) 50 μl iPSC-MSCs-CdM (iPSC-MSCs-CdM group, n = 12) at 4 sites of the left ventricle (LV). Three weeks after CdM injection, invasive hemodynamic measurements were performed. All mice were then sacrificed and detailed histological examination was performed to assess myocardial fibrosis, apoptosis and oxidative stress.

In order to verify the role of GDF-15 and MIF in iPSC-MSCs-CdM for the treatment of AIC, additional groups of mice with AIC were randomized to receive intramyocardial injection of 1) phosphate-buffered saline (Dox group, n = 12); 2) 50 μl iPSC-MSCs-CdM (iPSC-MSCs-CdM group, n = 12) or 3) 50 μl iPSC-MSCs-CdM with depletion of MIF and GDF-15 (iPSC-MSCs-CdM-MIF/GDF-15(−) group, n = 12) 4) 50 μl MIF/GDF-15 (MIF/GDF-15 group, n = 12) at 4 sites of the left ventricle (LV). The concentrations of MIF and GDF-15 mixture were the same as those in iPSC-MSCs-CdM. Briefly, in iPSC-MSCs-CdM, we first measured the concentration of GDF-15 was 4113 ± 121 pg/mg and MIF was 12316 ± 1244 pg/mg. Total protein concentration of iPSC-MSCs-CdM was 1.09 mg/ml. Then the concentration and total amount of GDF-15 and MIF in 50 μl of iPSC-MSCs-CdM were able to be calculated. Next we made concentrations of GDF-15 at 4.48 ng/ml and MIF at 13.42 ng/ml in ddH_2_0 solution to meet the same concentrations of GDF-15 and MIF presented in iPSC-MSCs-CdM. The total amounts of GDF-15 (0.224 ng) and MIF (0.67 ng) in 50 μl were used for injection. Human MIF (Catalog.300-69) and GDF-15 (Catalog.120-28) recombinant cytokines were purchased from PeproTech. Three weeks later, hemodynamic measurements were performed, and then all mice were sacrificed for histological study.

### Cardiac function assessment

At 3 weeks after CdM injection, mice were anesthetized (intra-peritoneal injection of 100 mg/kg of ketamine and 20 mg/kg of xylazine) to undergo transthoracic echocardiography and invasive hemodynamic assessment. M-mode echocardiogram was performed to measure LVEF and FS as previously described^55^. A 1.4-Fr high-fidelity microtip catheter connected to a pressure transducer (Millar Instruments, Houston, TX, USA) was inserted into the LV cavity via the internal jugular artery to evaluate LV pressure and dP/dt using the PowerLab system (AD Instruments, Inc., Colorado Springs, CO, USA)^55^. In this study, the echocardiographic and hemodynamic analyses were performed by an experienced investigator who was blinded to the experimental information.

### Histological analysis

All mice were sacrificed following hemodynamic study; hearts were harvested and fixed with 10% buffered formalin and embedded in paraffin, then sectioned to 5 μm slides. HE staining and Sirius red staining were performed. The ratio of fibrotic area was quantified with 6 randomly chosen high power fields for each heart section, 6 mice for one group and analyzed using Image J with additional threshold color plug-in to process the file images.

### MDA assay

The concentration of MDA in the heart tissues and treated NRCMs from different experimental groups was measured using the Thiobarbituric Acid Reactive Substance Assay Kit (Cayman Chemical), according to the manufacturer’s instructions.

### Statistical analysis

Values are expressed as mean ± standard deviation. The significant differences between groups were analyzed with unpaired Student *t* test for two groups or one-way ANOVA, followed by Bonferroni test for more than 2 groups. A value of *P* *<* 0.05 was considered statistically significant.

## Additional Information

**How to cite this article**: Zhang, Y. *et al*. Potent Paracrine Effects of human induced Pluripotent Stem Cell-derived Mesenchymal Stem Cells Attenuate Doxorubicin-induced Cardiomyopathy. *Sci. Rep*. **5**, 11235; doi: 10.1038/srep11235 (2015).

## Figures and Tables

**Table 1:** Top 10 secreted factors over-represented in iPSC-MSCs.

Secreted factors	**mRNA Refseq**	**P-value**	**Mean ratio (iPSC-MSCs/BM-MSCs)**	**Involved biological process**
MIF	NM_002415	0.0007	9.0226	Inflammatory response, apoptosis, cell proliferation, oxidation
EMAP-II	NM_004757	0.0048	8.4005	Apoptosis, inflammatory response, cell migration, cell proliferation
GDF-15	NM_004864	0.0046	7.4638	inflammation, apoptosis, PI3K/AKT signaling, Angiogenesis, cardioprotective properties, tissue differentiation
Osteoprotegerin	NM_002546	0.0024	7.2562	Apoptosis, OPG/RANK/RANKL axis
PDGF-AA	NM_002607	0.003	5.0133	Response to endogenous stimulus, inflammatory response, cell migration, cell proliferation
FGF-16	NM_003868	0.0024	4.9466	Actin cytoskeleton, MAPK signaling pathway, glycoprotein, response to abiotic stimulus,
GFR alpha-4	NM_022139	0.005	4.1354	Lipoprotein, glycoprotein, signal
Thrombospondin-1	NM_003246	0.0029	3.9156	Apoptosis, cell migration, inflammatory response, cell proliferation
IL-24	NM_006850	0.0021	3.7026	Apoptosis
ErbB3	NM_001005915	0.0016	3.701	Apoptosis, response to endogenous stimulus, cell proliferation

**Table 2:** Top 13 secreted factors over-represented in BM-MSCs.

Secreted factors	**mRNA Refseq**	**P-value**	**Mean ratio (BM-MSCs/iPSC-MSCs)**	**Involved biological process**
MMP-15	NM_002428	0.024	42.55319	Phosphoprotein, transmembrane, glycoprotein, calcium ion binding, polymorphism, disulfide bond
Activin RII A/B	NM_001106	0.002	5.885815	Cell differentiation
EGF R (ErbB1)	NM_005228	0.0603	5.630631	Cell migration, cell proliferation, cell cycle, apoptosis
Activin A	NM_002192	0.0403	5.51572	mesoderm induction, cellular proliferation and differentiation, cell survival
AR (Amphiregulin)	NM_001657	0.03	4.512635	Cell proliferation, mitogen
CD30 Ligand	NM_001244	0.0328	3.318951	Cell proliferation, apoptosis
S100 A8/A9	NM_002965	0.0844	3.063725	Inflammatory response, cell migration
CCR7	NM_001838	0.0167	2.783964	Inflammatory response, cell migration of memory T cells
Glypican 3	NM_001164617	0.0585	2.45821	Cell division, cell differentiation
PDGF R alpha	NM_006206	0.0827	2.278943	Cell migration, cell proliferation
VEGF R2 (KDR)	NM_002253	0.069	2.198769	Cell migration, cell proliferation
SIGIRR	NM_021805	0.0641	2.042901	Inflammatory response
Chem R23	NM_001142345	0.0029	2.024701	Cell migration
